# A Circular

**Published:** 1885-05

**Authors:** J. Taft, C. W. Spalding, F. J. S. Gorgas


A CIRCULAR.—The periodical literature of the dental profession of this country has grown to such proportions as to call for joint action on the part ot those more prominently connected with the direction of its affairs.

Union and co-operation give quality and strength to any enterprise beyond what can be attained by individual effort. With this fact in mind it is suggested that a meeting of the editors in chief of the dental journals published in this country, or their representatives, especially of the monthlies whose subscription price is not less than two dollars a year, and of the quarterlies whose subscription price is not less than one dollar a year, be held at Minneapolis at the time of the meeting of the American Dental Association in August next, for the purpose of considering propriety and feasibility of organizing in some form with a view of promoting the interests of American dental journalism.

If this call meets with sufficient encouragement a time and place of meeting will be fixed and due notice given to all who may respond to this circular.

J. TAFT.
C. W. SPALDING.
F. J. S. GORGAS.

